# Protein and growth during the first year of life: a systematic review and meta-analysis

**DOI:** 10.1038/s41390-023-02531-3

**Published:** 2023-03-20

**Authors:** Gregorio P. Milani, Valeria Edefonti, Valentina De Cosmi, Silvia Bettocchi, Alessandra Mazzocchi, Marco Silano, Angelo Pietrobelli, Carlo Agostoni

**Affiliations:** 1 Pediatric Unit, Fondazione IRCCS Ca’ Granda Ospedale Maggiore Policlinico, Milan, Italy; 2 Department of Clinical Sciences and Community Health, Università degli Studi di Milano, Milan, Italy; 3 Unit of Human Nutrition and Health, Department of Food Safety, Nutrition and Veterinary Public Health, Istituto Superiore di Sanità, Rome, Italy; 4 Department of Surgical Science, Dentistry, Gynecology and Pediatrics, Pediatric Unit, Verona University Medical School, Verona, Italy; 5 Pennington Biomedical Research Center, LSU System, Baton Rouge, LA USA; 6 SC Pediatria-Immunoreumatologia, Fondazione IRCCS Ca’ Granda Ospedale Maggiore Policlinico, 20122 Milano, Italy

## Abstract

**Abstract:**

Dietary protein intake in the first year of life might influence later growth. We conducted a systematic review to investigate the growth effects of interventions based on infant formula composition providing different amounts of protein within the first year of life of healthy term infants; in the absence of other comparable information over the investigated period, a meta-analysis further compared weight or length gain at 120 days from high- (>2.0 g/100 kcal) and low-protein (≤2.0 g/100 kcal) content formula groups. Twelve papers (*n* = 2275) were included and five of them (*n* = 677) contributed to the meta-analysis. Most studies compared a high-protein formula, a low-protein formula, and breastfeeding. Evidence from the systematic review was inconclusive due to heterogeneity in design and treatments. In the presence of modest heterogeneity but in the absence of publication bias, the weighted mean difference for weight gain at 120 days was –0.02 g/day (95% CI: –1.41, 1.45); with higher heterogeneity, the weighted MD estimate of length gain at 120 days was 0.004 cm/month (95% CI: –0.26, 0.27). Although limited and underpowered, evidence from the meta-analysis does not support the assumption that high- vs. low-protein content formulas during exclusive milk-feeding lead to different growth outcomes in the first months of life. Prospero registration number: CRD42017058535.

**Impact:**

- The optimal amount of dietary protein that should be given to healthy full-term infants early in life is still debated.
- Despite heterogeneity in study design, treatments, and outcomes, this systematic review showed that there is no clear-cut effect on the growth of different amounts of protein intake from formulas or complementary feeding.
- Evidence from the meta-analysis based on the five articles enrolling infants <1 month of life does not support the previous assumption that high- vs. low-protein content formulas during exclusive milk-feeding lead to different growth outcomes in the first 4 months of life.

## Introduction

The role of nutritional programming in infancy on later growth has been widely debated in recent years. Recently, a group of expert committees systematically reviewed the levels of evidence underlying guidelines for a wide variety of infant feeding practices and related health outcomes within the Pregnancy and Birth to 24 Months Project. For almost all of the research questions, the committees concluded that the level of evidence supporting single outcomes was low to modest, at best.^1^ One important issue in infant feeding concerns the optimal level of dietary protein that should be given to infants. Protein intake in the first year of life is, indeed, considered one of the main determinants of growth later in life.^2^ In this period, an insufficient protein intake may lead to detrimental consequences, such as growth failure and altered body composition and metabolism.^3^ On the other hand, previous literature suggests that a high-protein intake earlier in life is associated with a higher weight gain later on.^4–6^ Indeed, breastfed infants, with a limited protein intake and a balanced energy-to-protein ratio supplied by human milk, show a lower risk of developing fatness later in life. However, the evidence is still controversial, and the underlying mechanisms are unclear.^7–10^ In more recent papers, the suggestion of an association between earlier protein intakes and later fatness has been supported by several authors on the basis of heterogeneous studies, including randomized clinical trials (RCTs).^11,12^ Finally, a quantitative synthesis of the evidence via meta-analysis is currently not available on the effects of different amounts of protein intake within the first year of life.

The current paper is aimed at investigating the effects on growth outcomes of different amounts of protein intake in healthy full-term infants within the first year of life through: (1) a systematic review of the literature that includes any interventions with different protein content (e.g., infant formulas, follow-on formulas, or complementary feeding); and (2) a quantitative synthesis of the evidence via a meta-analysis that considers the effect of different formula-based interventions on growth outcomes consistently measured at any timepoints in similarly designed studies.

## Materials and methods

### Systematic review

A systematic review of the literature was initially performed in June 2018 and then updated on October 15, 2021. The search was carried out via PubMed (www.ncbi.nlm.nih.gov/pubmed), Embase (www.emabase.com), Web of Science (www.isiknowledge.com), and the Cochrane library (www.cochranelibrary.com), following PRISMA guidelines.^13^ The following search terms were used: (first year OR first year of life OR first months OR first months of life) & (dietary protein) & (growth OR length OR weight OR body mass index OR skinfold thickness). Only human studies reported in English were considered. Letters to the editor, abstracts, and proceedings from scientific meetings were excluded from the analysis.

### Included studies

We included in this systematic review any RCT on healthy term infants that: (1) compared at least two arms presenting different amounts of dietary protein in the first year of life and (2) evaluated their short- (i.e., ≤1 year from the beginning of the intervention) or long-term (i.e., >1 year from the beginning of the intervention) effects on growth. Possible interventions include infant formulas, follow-on formulas, or complementary foods. Among possible short- or long-term growth outcomes, we considered body length, weight, body mass index (BMI), waist circumference, and body fat content. We did not consider studies including mixed-type interventions, where protein intake was targeted together with additional dietary components (e.g., amino acids supplementation). Studies that did not allow to separate out the effects of protein intake in the first year of life from that of later periods were excluded. In addition, we excluded studies that did not allow us to assess arm-specific dietary protein intake. Studies targeting selected populations following specific research questions (e.g., studies selecting a priori infants born from overweight mothers only) were also discarded. Two authors (V.D.C., A.M.) independently selected the articles, retrieved and assessed the potentially relevant ones. Discrepancies in the articles’ selection or disagreement on the interpretation of methods or results were resolved by a face-to-face discussion; if the discrepancy persisted, a third researcher was consulted (C.A.).

### Data collection, extraction, and analysis

Pairs of review authors (G.P.M., V.E., A.M., S.B.) independently extracted the data. Controversies were resolved by discussing with the senior author (C.A.). From each included article, the following information was extracted and inserted in a structured database: (1) general characteristics of the studies; (2) study design, inclusion criteria, data collection occasions, and characteristics of the intervention; (3) outcome; (4) main results; and (5) strengths and limitations of the included studies.

When present, we also collected the main results on high- or low-protein arm vs breastfeeding.

### Study quality

All included articles were independently rated for quality by two researchers (G.P.M. and V.E.), using the Quality Assessment Tool for Clinical Trials from the NIH National Heart, Lung, and Blood Institute.^14^ If the ratings were different, a third author (C.A.) was consulted for quality adjudication. Each study was judged as being of “good,” “fair,” or “poor” quality. We did not identify a cut-off for the total score (calculated by summing up the 1s corresponding to the “yes” marks), but we carefully evaluated the “no” items to assess the overall risk of bias of the examined study.

### Meta-analysis

We focused our meta-analysis on the comparison between growth outcomes derived from formulas showing different amounts of protein. Restriction to formulas allows us to better control treatment-related heterogeneity, potentially due to the presence of a combination of formula and complementary feeding. Among the studies included in the systematic review, we selected those which enrolled all participants and started their intervention within the first month of life, to reduce as much as possible the effect of prolonged breastfeeding before switching to the formula intervention. In case of relevant missing information or to verify the reliability of the information, the corresponding author of the article was contacted by email. If the author did not respond to our query, a second attempt by email was performed at least 20 days later. If the second email went unanswered, missing data were imputed from those available in the report. Additional details were provided in the following.

### Included studies

We identified five studies to be included in the meta-analysis.^15–19^ These studies consistently measured growth outcomes at 120 days of life of the infants and were comparable in terms of treatment and potential treatment effects over the reference period. The study by Oropeza-Ceja et al.^16^ was included after contact with the corresponding author who confirmed that all infants assigned to the three formula groups were exclusively or mixed breastfed for no more than 30 days. Although three papers within the European ChildHood Obesity Project (CHOP)^20–22^ and the studies by Ziegler et al.,^23^ Åkeson et al.^24^ and Larnkjæ et al.^25^ considered formula-based interventions, the corresponding results were excluded from the meta-analysis because infants were enrolled up to approximately 2,^20–22^ 3,^23,24^ or 9^25^ months of life. In addition, we excluded the Picone et al. article because the outcome measure was not available at 4 months.^26^

#### Treatments

Treatments under comparison included a high- and low-protein content infant formula, but their exact specification varied across studies (Table 1). After inspecting study-specific cut-offs, we assumed that subjects receiving an amount of protein ≤2.0 g/100 kcal belong to the low-protein content formula group, whereas those receiving >2.0 g/100 kcal belong to the high-protein content formula group. One paper^16^ provided a four-arm design, where low-, medium-, and high-protein arms were considered, together with breastfeeding. In accordance with our definition, we included the original low- (1.4 g/100 kcal) and medium-protein (1.8 g/100 kcal) content groups into one low/medium-protein-content group of 35 participants. The mean and standard deviation of the growth outcomes were calculated using the weighted mean and the decomposition of the total variance in between- and within-groups variance (Supplementary Materials and methods—text).

**Table 1:** Characteristics of the studies presented in the papers included in the systematic review.

Reference Study type Setting	Sample size	Subjects per protocol analysis	Study design	Growth outcomes	Others outcomes	Main findings	Weaknesses/limitations	Strengths
Turck et al. (2006)^15^ Double-blind RCT (non-inferiority trial) Lille, Marseilles, Paris and Reim, France	*N* = 156 (50 high-protein IF; 51 low-protein IF; 55 BF)	*N* = 126 (50 high-protein IF; 51 low-protein IF; 25 BF)	Newborns (age <7 days), assigned to a low- (1.8 g/100 kcal) or high-protein (2.6 g/100 kcal) content formula for 120 days Measurements (weight and length) performed at enrollment, after 15, 30, 60, 90 and 120 days Median age at enrollment: 4.2 days in high-protein IF, 4.4 in low-protein IF and 4.4 in BF	Daily weight gain between enrollment and 120 days (primary outcome) Gains in weight, length and BMI (secondary outcomes) at 15, 30, 60, 90 and 120 days		The difference in mean daily weight gain from enrollment to 120 days between high- and low-protein content formula was +0.38 g/day, with a 95% confidence interval of –2.59 to +1.83 g/day There was no difference between the two formula groups with regard to secondary outcomes at any of the time intervals considered	Only 36% of infants in BF group were exclusively breastfed for 120 days	Intervention was started in the first week of life Infants received mother’s milk or IF up to the 4 months of life Formulas casein/whey ratio was changed (casein/whey ratio of 70/30 in high-protein IF; casein/whey ratio of 30/70 in low-protein IF) Sample size was calculated to detect effects on body weight gain of different IF protein contents Very low rate of drop-out
Oropeza-Ceja et al. (2018)^16^ Single-blind RCT Queretaro, Mexico	*N* = 308 (42 high-protein IF; 24 medium-protein IF; 30 low-protein IF; 212 BF)	*N* = 141 (24 high-protein IF; 18 medium-protein IF; 17 low-protein IF; 82 BF)	Infants ≤40 days of age assigned to low- (1.0 g/100 ml), medium- (1.3 g/100 ml) or high-protein (1.5 g/100 ml) content formula from 30 to 120 days of life Measurements (weight, length and BMI) performed at enrollment and after 30 and 120 days Median age at enrollment: 22 days in high-protein IF, 19 in medium-protein IF, 19 in low-protein IF and 21 in BF	Weight and length gain between 30 and 120 days		Infants fed with low-protein IF had a weight gain and a weight-for-age *Z*-score (25.8 g/day; –0.6 *Z*-score) similar to BF infants (27.0 g/day; –0.4 *Z*-score) and significantly lower than the medium-protein content (*p* = 0.031) and the high-protein content (*p* = 0.014) groups There were no significant differences in length gain at 120 days, weight-for-length *Z*-score, length-for-age, BMI *Z*-score among the groups	Some infants were enrolled after the first week of life IFs were not isocaloric High drop-out rate	Infants received mother’s milk or IF up to the 4 months of life Formulas casein/whey ratio was changed (casein/whey ratio of 40/60 in high-protein IF; casein/whey ratio of 35/65 in medium- and low-protein IF) Sample size was calculated to detect effects of different IF protein contents on weight gain
Putet et al. (2016) (Early Protein and Obesity in Childhood, EPOCH study)^17^ Double-blind RCT Lyon, France	*N* = 238 (80 high-protein IF; 74 low-protein IF; 84 BF)	*N* = 210 (74 high-protein IF; 71 low-protein IF; 65 BF)	Newborns (age <7 days) assigned to a low- (1.8 g/100 Kcal) or high-protein (2.7 g/100 Kcal) content formula from birth to 120 days Measurements (weight and length) performed at 2 weeks and 2, 4, 6, 9, 12, 36, 48 and 60 months. Body composition (air-displacement plethysmography at 6 months of age or younger; dual energy X-ray absorptiometry at 12, 36 and 60 months of age) was measured at 2 weeks and at 4, 6, 12, 36 and 60 months of age. The skinfold thickness (triceps, biceps, sub-scapular and abdominal) and mid-arm circumference were measured at 36, 48 and 60 months of age Median age at enrollment: 3.3 days in high-protein IF, 3.1 in low-protein IF and 3.2 in BF	Weight, length, BMI and body composition at 2 weeks and 2, 4, 6, 9, 12, 36, 48 and 60 months	Plasma IGF-1 concentrations at 4 months (primary outcome) IGF-1 concentrations at 2 weeks and 9 months of age; postprandial insulin, C-peptide, IGF-binding protein (IGFBP)-2, IGFBP-3 and glucose concentrations at 2 weeks, 4 and 9 months of age	During the first 60 months of life, weight, length and BMI were almost always similar in high- and low-protein IF. Body weight at 4 and 6 months and length at 9, 12 and 36 months were lower in low-protein IF The fat mass and fat-free mass were similar in the IF groups during the first 60 months of life	Sample size was not calculated to detect effects of different IF protein contents	Intervention was started in the first week of life Infants received mother’s milk or formula up to the 4 months of life Formulas casein/whey ratio was changed (casein/whey ratio of 30/70 in high- and low-protein IF)
Fleddermann et al. (2014) (The BeMIM study: Belgrade-Munich Infant Milk Trial)^18^ Double-blind RCT (non-inferiority study) Belgrade, Serbia	*N* = 398 (106 high-protein IF; 107 low-protein IF; 185 BF)	*N* = 256 (82 high-protein IF; 82 low-protein IF; 92 BF)	Infants (recruited until the age of 28 days) assigned to a low- (1.89 g /100 kcal) or high-protein (2.30 g/100 kcal) content formula from 30 to 120 days of life Measurements (weight and length) performed at baseline and at 30, 60, 90 and 120 days Median age at enrollment: 19 days	Weight and length gain between 30 and 120 days and at 60 and 90 days of life		No differences between IF groups for weight gain from 30 to 120 days of age Length gain was higher in low-protein IF (0.11 ± 0.02 cm/day) compared to high-protein IF group (0.10 ± 0.02 cm/day, *p* = 0.02) Weight and length gains in BF infants were significantly lower than in IF infants	Some infants were enrolled after the first week of life High drop-out rate	Infants received mother’s milk or formula up to the 4 months of life Formulas casein/whey ratio was changed (casein/whey ratio of 40/60 in high- and low-protein IF) Sample size was calculated to detect effects on weight gain of different IF protein contents
Liotto et al. (2018)^19^ Single-blind RCT Milan, Italy	*N* = 168 (58 high-protein IF; 55 low-protein IF; 55 BF)	*N* = 150 (50 high-protein IF; 50 low-protein IF; 50 BF)	Newborns (age ≤21 days) assigned to a low- (1.9 g/100 kcal) or high-protein (2.5 g/100 kcal) content formula for the first 120 days of life Measurements (weight and length) and body composition (by air-displacement plethysmography) assessed at enrollment, 60 and 120 days of life Median age at enrollment: 5.3 days	Weight gain (primary outcome), weight, length, fat mass and fat-free mass (secondary outcome), at 60 and 120 days of life	Gastrointestinal symptoms at 60 and 120 days of life (secondary outcomes)	Weight gain, weight and length were similar between the 2 IF groups at each study point IF groups showed similar fat mass deposition at each study point compared to the BF group, whereas a higher amount of fat-free mass of IF infants was detected	Some infants were enrolled after the first week of life IFs were not isocaloric Information on whey/casein ratio not reported	Infants received mother’s milk or formula up to the 4 months of life Sample size was calculated to detect effects on weight gain of different IF protein contents
The European Childhood Obesity Project (CHOP) Double-blind RCT Belgium, German, Italy, Poland and Spain			Infants (age <56 days) assigned to a low- (1.77 g/100 kcal) or high-protein (2.9 g/100 kcal) content formula for ~5.5 months. Then, infants were ri-assigned to a low- (2.2 g/100 kcal) or high-protein (4.4 g/100 kcal) content follow-on formula up to 12 months of life				Some infants were enrolled after the first week of life and recruitment lasted up to 2 months No change of the casein/protein ratio Follow-on formula with protein content equivalent to unmodified cow’s milk High drop-out rate In some subjects weight and length were obtained by a telephone interview	The overall length of the interventions (infant plus follow-on formulas) was up to the end of the 12th month of life Infants received mother’s milk or formula up to the 4 months of life Switch from infant to follow-on formula Length of follow-up Sample size was calculated to detect effects of different IF protein contents on length
Koletzko et al. (2009)^20^	*N* = 1727 (574 high-protein IF, 564 low-protein IF, 589 BF)	*N* = 933 (322 high-protein IF; 313 low-protein IF; 298 BF)	Median age at enrollment: 15 days in high-protein IF, 16 days in low-protein IF and 12 days in BF	Weight and length at 3, 6, 12, and 24 months (primary outcomes)		Weight in *Z*-scores was higher in high-protein IF group at 3, 6 and 12 months as compared with low-protein formula IF BMI was higher in high-protein IF group at 6 and 12 and 24 months as compared with low-protein formula IF. At 24 months, no difference was observed for length and weight The weight-for-length *Z*-score of infants was lower in the low-protein IF at 6 and 12 months only. At 24 months, the weight-for-length *Z*-score of infants in the low-protein IF group was 0.20 (0.06, 0.34) lower than that of the high-protein IF group and did not differ from that of the BF group		
Weber et al. (2014)^22^		*N* = 657 (221 high-protein IF; 227 low-protein IF; 209 BF)		Weight, length, and BMI from 2 to 6 years of age every 6 months		Weight was slightly higher (0.5 kg) in high-protein IF group at 6 years Height at 6 years was similar in both IF groups BMI in the high-protein content IF group was 0.51 kg/cm^2^ higher (*p* = 0.009) at 6 years of age, and the risk of obesity was >2 times higher (*p* = 0.024)		
Escribano et al. (2012)^21^ (subset of CHOP infants from Germany and Spain)		*N* = 66 (17 high-protein IF; 24 low-protein IF; 25 BF)		Weight, length, BMI, and weight gain velocity at 2 days, 6 and 12 months of life, and fat mass (total, *Z*-score, and index), fat-free mass (total, *Z*-score, and index) at 6 months of life		Weight gain velocity was higher in high- than in low-protein IF group (807.8 ± 93.8 vs 724.2 ± 110.0 g per month, *p* = 0.015) Weight was higher in high- as compared to low-protein IF group (8.20 ± 0.71 vs 7.60 ± 0.75 kg). No difference was observed in relation to length at 6 months between IF groups Weight-for-length was higher in high- as compared to low-protein IF group at 6 months (0.117 ± 0.011 vs 0.114 ± 0.011 kg/cm) and at 12 months (no mean values reported in the text) BMI was higher in high- as compared to low-protein IF group at 6 months (18 ± 1.45 vs 16.68 ± 1.30 kg/m^2^) and 12 months (no mean values reported in the text) No difference was observed in relation to fat or fat-free mass measures between the two IF groups		
Ziegler et al. (2015)^23^ Double-blind RCT Iowa and Oklahoma, United States of America	*N* = 306 (97 high-protein IF; 97 low-protein IF; 112 BF)	*N* = 279 (87 high-protein IF; 87 low-protein IF; 105 BF)	Infants (3 months of age) assigned to a low- (1.61 g/100 kcal) or high-protein (2.15 g/100 kcal) content formula up to 12 months of life Complementary foods were allowed in small amounts from 4 to 6 months and unrestricted after 6 months of age Median age at enrollment: 3 months	Weight gain between 3 and 6 and up at 12 months of age. Monthly (up 5.5 months of life) and bimonthly (up to 12 months of life) measurements (primary outcome)	Albumin, blood urea nitrogen, IGF-1 was determined at 83, 168, and 360 days (secondary outcomes)	Weight gain from 4 to 12 months was slightly lower (*p* < 0.03) in low- vs high-protein content formula and was higher in both IF groups as compared to BF group (*p* < 0.0001) The odds ratio for a weight >85th percentile was 0.40, 95% CI 0.18–0.89) for low- vs high-protein content formula	Some infants were enrolled after the first week of life and recruitment lasted up to 3 months Complementary feeding not well defined The slight weight difference among groups from 4 to 12 months is not detailed High-protein formula had a unmodified whey/casein ratio	Infants received mother’s milk or formula up to the 4 months of life Sample size was calculated to detect effects of different IF protein contents on weight gain
Åkeson et al. (2000)^24^ RCT, information on blindness not reported, Sweden and Italy	139 (number of infants attributed to different formula-based groups not reported)	57 (17 high-protein IF, 18 medium-protein IF and 17 high-protein IF)	Infants (3 months of life) assigned to an IF with 13, 15 or 18–20 g/l (18 for Swedish and 20 for Italian infants) protein concentration up to 12 months of life Measurements (weight and length) assessed at enrollment (3 months), 4, 6, and 12 months Seven-day dietary record Median age at enrollment: not reported	Weight and length gain at 3–6 months and at 6–12 months in Swedish and Italian infants	Plasma albumin, urea, and amino acids Protein and energy intake at 6 and 12 months	There was no difference in weight and length gain between Swedish and Italian infants consuming formulas with the same concentration	No sample size calculation No change of the casein/protein ratio across IFs (18/82) No details on energy intake of the 3 IFs at 3 months Very complicated design, with 3 IFs, weaning foods, and dietary habits of 2 countries under comparison	Only infants that had been breastfed for at least 2–3 months were included Infants breastfed at 6 months and beyond were excluded
Larnkjær et al. (2009)^25^ RCT, information on blindness not reported, Denmark	*N* = 94 (46 whole milk vs 48 low-protein IF)	*N* = 83 (38 high-protein IF; 45 low-protein IF)	Infants (9 months of age) assigned to a low-protein content formula (i.e., any IF available from the market which had ≥1.1 to ≤1.5 g/100 ml in general and mostly 1.2 or 1.5 g/100 ml) or (high-protein content) whole milk up to 12 months of life Further receiving or not a daily fish oil supplement within a 2 times 2 factorial design Fish oil supplementation had no effect on any of the outcomes Measurements (weight, length, increase in weight and length) assessed at enrollment (9 months) and at the end of the study (12 months) Seven day dietary record Median age at enrollment: not reported	Weight and length at 12 months of age	IGF-1, IGFBP-3 Protein (% of energy intake) and energy intake at 9 and 12 months	There was no difference in weight and length between the two groups at 12 months of life	Within the low-protein content formula group, different protein content formulas were admitted Power calculation not clear IFs were not isocaloric Information on whey/casein ratio not reported	
Picone et al. (1989)^26^ Blind RCT, no additional information on blindness, Italy	*N* = 43 (10 low-protein IF, 10 medium-protein IF, 12 high-protein IF, 11 BF)	*N* = 40 (10 low-protein IF; 10 medium-protein IF; 10 high-protein IF, 10 BF)	Newborns (at birth, in hospital, as first feeding) assigned to an IF with 11.2, 13.1 or 14.8 g/l protein concentration for 3 weeks Measurements (weight, length, head circumference) assessed at enrollment (birth) and at 2, 4, 8, and 12 weeks Food record for formula consumption Median age at enrollment: not reported	Weight and length at 2, 4, 8 and 12 weeks of age	Plasma albumin, prealbumin, urea and amino acids, urinary amino acids Protein and energy intake at weeks 4, 8 and 12	There was no difference in weight and length among the three groups at any of the four timepoints measured	No power calculation No change of the casein/protein ratio	Intervention was started in the first week of life Low drop-out rate

*RCT* randomized clinical trial, *IF* infant formula, *BF* breastfeeding, *IGF* insulin growth factor, *WAZ* weight for age *Z*-score, *LAZ* length for age *Z*-score.

#### Outcome measures

We conducted two parallel meta-analyses considering as the primary outcomes weight gain at 120 days of life and length gain at 120 days of life, respectively. After contacting the corresponding authors, available information was still insufficient (two studies only) to carry out a meta-analysis on BMI gain at 120 days. When the standard deviation of weight or length gain was not reported and it was not possible to calculate it from different data in the paper (for weight gain in one study^17^ and for length gain in two studies^17,19^), we imputed it as the mean of the arm-specific standard deviation provided by the remaining studies included in the meta-analysis. Sensitivity analyses were later conducted to assess the effect of this strategy, by varying the imputed standard deviations from the minimum to the maximum values available in each arm.

### Statistical analysis

We used the mean difference (MD) in each growth outcome as the measure of treatment effect. When adjusted estimates of the intervention effect, together with their standard errors, were derived from an analysis of covariance model in the original studies,^16^ they were combined with the arm-specific change-from-baseline scores on the MD scale, as they give the most precise and least biased estimates of intervention effects. We calculated the summary estimates of the weighted MD using both fixed-effects (based on the inverse variance method) and random-effects (based on the DerSimonian and Laird estimation method) models.^27,28^ The forest plot presented study-specific and combined estimates of the MDs from random-effects models, to provide more conservative estimates.^29^

Statistical heterogeneity among studies was assessed via *χ*^2^ test on the Q statistic (corresponding *p* value < 0.10 suggests the presence of heterogeneity);^28^ the presence of potential inconsistencies was quantified through the *I*^2^ statistic^30^ (*I*^2^ statistic < 25%: low heterogeneity; 25% < *I*^2^ statistic < 75%: moderate heterogeneity; *I*^2^ statistic > 75%: high heterogeneity). For each trial, we plotted the treatment effect by its standard error within the funnel plot. Besides visual inspection, the presence of symmetry in the funnel plot was assessed with Egger’s and Begg’s tests, to assess if the effect decreased with sample size increasing.^31,32^

A cumulative meta-analysis was performed to assess how the overall estimate changes as each study is added to the pool of the previously published studies; an influence analysis excluded each study at a time from the meta-analysis. The limited number of studies included in the meta-analysis did not allow to carry out the subgroup analyses by grade and type of sponsorship originally planned in the protocol.

We also calculated the post hoc power of each meta-analysis under fixed or random-effects models by assuming the expected effect size (Cohen’s *d*), sample size, number of studies, and degree of heterogeneity to be those identified in our meta-analyses. In detail, we used the sum of the two arm-specific medians as the (total) sample size; we re-expressed the absolute (unstandardized) MD between the two arms in terms of effect size, as measured by Cohen’s *d*. To provide a fair comparison, we also calculated the post hoc study-specific power from a two-sided independent samples *T* test with the single study-specific sample sizes and effect sizes (Cohen’s *d*), as well as an alpha level equal to 0.05. All the statistical analyses were performed using STATA software (version 13; StataCorp, College Station, TX).

### Risk of bias assessment

Risk of bias was assessed by two authors (G.P.M. and V.E.) for all studies included in the meta-analysis according to the criteria presented in the Cochrane Handbook for Systematic Reviews of Interventions (Version 5.1.0).^33^

## Results

### Systematic review

**Fig. 1:**
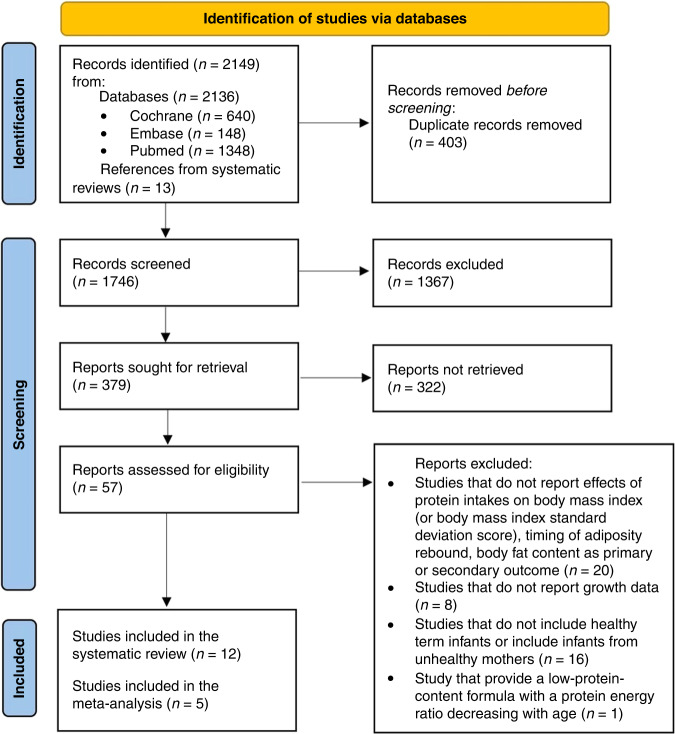
Flowchart of the study selection process. Flowchart of the study selection process for the systematic review and meta-analysis following the guidelines from the Preferred Reporting Items for Systematic Reviews and Meta-Analyses (PRISMA) group.

The search process is shown in Fig. 1. A total of 12 papers^15–26^ corresponding to 10 RCTs (2275 participants) mostly from Europe and the United States were retained for the systematic review (Table 1). Two papers^21,22^ (723 participants) provided a follow-up of the previously published CHOP study.^20^ Five were double-blind trials, two^16,19^ single-blind ones and in one study^26^ blindness was present but not described in detail. Trials considered eligible infants those recruited at birth,^26^ or aged <7,^15,17^ ≤21^19^ or 28,^18^ ≤40 days of life^16^ or <56 days of life;^20–22^ the remaining studies enrolled infants of 3^23,24^ and 9^25^ months of life.

Most studies compared a high-protein formula, a low-protein formula, and breastfeeding. Oropeza-Ceja et al. and Picone et al.^16,26^ considered high- (or formula 15^26^ or 2.14 g/100 kcal), medium- (1.8 g/100 kcal^16^ and formula 13^26^ or 1.85 g/100 kcal), and low-protein (1.4 g/100 kcal^16^ and formula 11^26^ or 1.57 g/100 kcal) formulas, as well as breastfeeding; due to its focus on total protein intake (including weaning foods), Åkeson et al.^24^ enrolled breastfed infants of 3 months and assigned them to high- [formula 18 (i.e., 2.57 g/100 kcal) or 20 (i.e., 2.85 g/100 kcal) depending on the group], medium- (formula 15 or 2.14 g/100 kcal), and low-protein (formula 13 or 1.85 g/100 kcal) formulas, which, however, were higher in protein concentration than in Picone et al.^26^ study. In one article,^25^ infants fed with any infant formulas available from the market were included in the formula group. The number of subjects was similar across arms (i.e., maximum difference between pairs of arms ≤20%) in most of the papers.^15–18,22,24–26^

The minimal set of anthropometric variables collected in all studies included body weight and length. The time-schedule of data collection differed across papers. Although all studies except one^17^ reported anthropometric measurements at enrollment, some studies treated infants and checked anthropometric measures on the same time span^15,16,18,19,21,24–26^ and others monitored anthropometric measures on a longer follow-up (e.g., several years after the intervention).^17,20,22^ In either case, the most common intervention strategy considered treatment from enrollment to 120 days of life of the infant. However, heterogeneity was present even when considering age at enrollment within the first month of life.^15–19^ This implied that, while some studies were likely to actually treat infants for about 4 months in total,^15,17^ one study^18^ treated infants for 3 months, another two^16,19^ were likely to include subsets of older infants, treated for less than 4 months. In addition, within the same period of 120 days of an infant’s life, the number and schedule of data collection differed across studies, with measurements taken 2,^19^ 3,^16,17^ 5,^18^ and 6^15^ times. Among selected articles, 8^15,17,18,20–23,26^ (67%) were of “good”, 3^16,19^ (25%) of “fair”,^25^ and one (8.3%) of “poor” quality.^24^ Two studies found a similar weight gain in the first 120 days for high- and low-protein content formulas.^15,19^ On the contrary, one study found a lower weight gain among infants in the low-protein, as compared with the high-protein formula group,^18^ and another study^16^ observed a lower weight gain in the low-protein formula group, as compared with medium- or high-protein content groups in the first 120 days of life. In addition, gain in weight was similar between Swedish and Italian infants assuming formulas with high-, medium-, and low-protein content over a 3–6-month and a 6–12-month period.^24^

Weight was similar between high- and low-protein content formulas in 4 papers, at 120 days of life,^19^ at 1 year,^25^ at 2 years,^20^ and at 5 years;^17^ however, it was found to be lower among infants in the low-protein formula group at 120 days of life in one paper^17^ and at 6 months in another paper.^21^ No significant differences in weight were also observed at 2, 4, 8 and 12 weeks in the comparison between low-, medium-, and high-protein content formulas in another article.^26^ Finally, at 6 years of life, weight was found to be slightly higher (3%) in the high-protein, as compared to the low-protein formula group.^22^

In two studies, a similar length gain in the first 120 days of life was detected.^15,16^ Another study found a higher (10%) length gain in the first 120 days of life in the low-protein content group.^18^ Finally, gain in length was similar between Swedish and Italian infants assuming formulas with high-, medium-, and low-protein content over a 3–6-month and a 6–12-month period of time.^24^

Length was similar between high- and low-protein content groups at 3^20^ and 6 months,^20,21^ 1,^20,25^ 2,^20^ 5^17^ and 6^22^ years of life. Similarly, no differences were observed at 2, 4, 8 and 12 weeks in the comparison between low-, medium-, and high-protein content formulas in another article.^26^ Weight for length was not different across the different protein content formulas at 120 days^16,20^ in one paper, but did differ in another one, being higher in the high-protein content formula group at 6 and 12 months.^20^

The BMI gain in the first 120 days was similar in two studies for low- vs. high-^15,16^ and medium-protein content formulas.^16^ One trial found that BMI was higher by 8%, 2% and 3% in high- vs. low-protein formula group at 6 months, 2 and 6 years of life, respectively.^20–22^ One study did not detect any difference in BMI between high- and low-protein content formula groups at 5 years of age.^17^

Among studies investigating differences in body composition in the high- and low-protein content formula groups, two observed that fat-free mass was similar at 120 days^19^ and at 5 years,^17^ and another one confirmed this lack of difference in fat-free mass, as well as in total fat mass, for infants of 6 months of age.^21^

### Meta-analysis

**Fig. 2:**
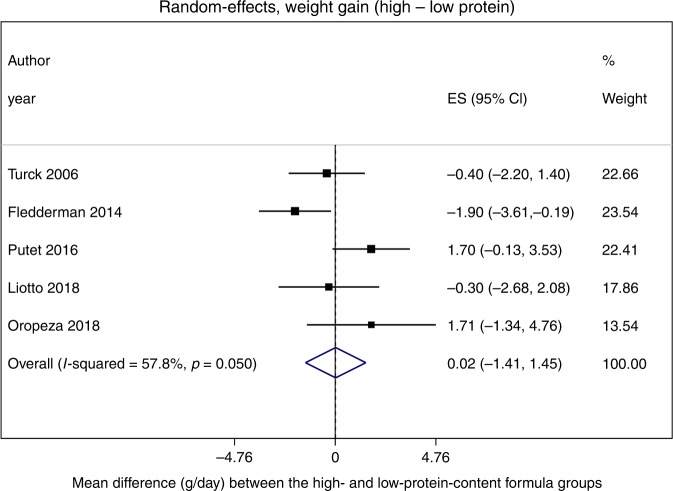
Forest plot showing the study-specific and pooled estimates of the effect on weight gain of high- vs. low-protein intakes in infant formulas. Results were derived from a random-effects meta-analysis where the measure of treatment effect was the mean difference in each growth outcome and displayed in the following way: (1) a square represented the study-specific point estimate of the intervention effect; (2) a horizontal line represented the precision of the point estimate in the form of the confidence interval. The area of the square reflected the contribution of each study to the meta-analysis in the form of weight. The pooled effect estimate and its confidence interval were represented by a diamond.

**Fig. 3:**
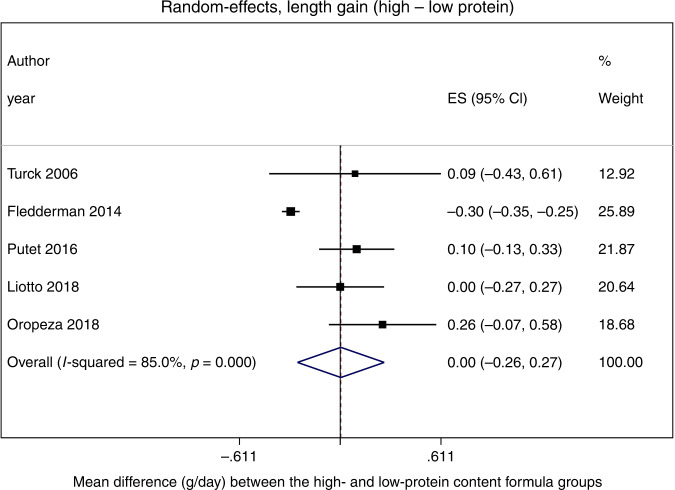
Forest plot showing the study-specific and pooled estimates of the effect on length gain of high- vs. low-protein intakes in infant formulas. See Fig. 2 for additional details.

Figures 2 and 3 show the study-specific and pooled estimates of the effect on weight and length gain of high- vs. low-protein intakes in infant formulas. The pooled MD of weight gain was 0.02 g/day (95% CI: –1.41, 1.45); the *p* value from the *χ*^2^ test on the *Q* statistic was equal to 0.05, and *I*^2^ suggested that 58% of the total variation was due to heterogeneity between studies (Fig. 2). In addition, the meta-analysis on length gain showed a pooled MD estimate of 0.004 cm/month (95% CI: –0.26, 0.27); the *χ*^2^ test on the *Q* statistic suggested the presence of heterogeneity between studies (*p* value < 0.001), and *I*^2^ ~85% (Fig. 3). For weight gain, the funnel plot did not show meaningful asymmetry of the studies (Supplementary Fig. 1). However, for length gain, two trials fell outside the funnel (Supplementary Fig. 2). Egger’s (*p* = 0.47) and Begg’s (*p* = 0.22) tests confirmed that there is no evidence of publication bias for weight gain. For length gain, the preferred Egger’s (Intercept: 3.09, 95% CI: 0.89–5.28, *p* = 0.02) test may suggest the presence of publication bias, whereas the less reliable Begg’s test provided inconsistent results. However, the few studies included in the meta-analysis had likely made the power of Egger’s and Begg’s tests too low to distinguish chance from real asymmetry.^33^ In addition, the cumulative meta-analyses showed that the overall estimates were stable as far as more recent studies were added to the pool of the previously published ones; the corresponding CIs always included 0.

For both growth outcomes, the influence analysis provided reassuring results. Indeed, when omitting one study at a time (1) the CIs of the five combined estimates always included 0, thus providing the same conclusion of the main analysis and (2) the five combined estimates obtained omitting each study at a time were included in the CI of the combined estimate based on all the studies (data not shown).

For each outcome measure, the four sensitivity analyses assessing a potential role of the imputed missing standard deviations provided reassuring results: the overall point estimates and 95% Cls were similar to those from the main analysis, with the CIs still including 0.

Post hoc power was extremely low for both meta-analyses: within the random-effect model, calculation with: number of studies = 5, total sample size = 100 (50 per arm), alpha = 0.05, type of test = two-sided, *I*^2^ = 58% for weight gain and *I*^2^ = 85% for length gain, effect size (Cohen’s *d*) = 0.0863 for weight gain and 0.0674 for length gain led to a power to detect an MD of 15% for weight gain and 13% for length gain. This is in line with results from study-specific power calculation, where power ranges from 6%^19^ to 72%^17^ for weight gain, with a median of 7%, and from 5%^19^ to 100%,^18^ with a median of 6%, for length gain (data not shown).

Most of the included studies showed a low risk of bias (Table 2).

**Table 2:** Risk of bias assessment for the studies included in the meta-analysis.

	Random sequence generation	Allocation concealment	Blinding of participants and personnel	Blinding of outcome assessment	Incomplete outcome data	Selective reporting
Turk et al. (2006)^15^	Low	Low	Low	Low	Low	Low
Oropeza-Ceja et al. (2018)^16^	Low	Low	Unclear	Unclear	Low	Low
Putet et al. (2016)^17^	Low	Low	Low	Low	Low	Low
Fledderman et al. (2014)^18^	Unclear	Low	Low	Low	Low	Low
Liotto et al. (2018)^19^	Low	Low	Unclear	Unclear	Low	Low

## Discussion

We conducted a literature analysis to gauge the hypothesis low- and high-protein content diets are associated with different growth outcomes by limiting the intervention timeframe to 1 year of life. Moreover, we carried out a meta-analysis that concentrates on studies as similar as possible, to reach sound conclusions. The systematic review shows that there is no clear-cut effect on the growth of different amounts of protein intake from formulas or complementary feeding during the first year of life of full-term infants. In addition, our meta-analysis points out that a low amount (≤2.0 g/100 ml) of proteins in formula milk has no significant effect on weight or length gain at 120 days, as compared to a high-protein content (>2.0 g/100 ml) formula.

Previous literature found a potential beneficial effect, sometimes modest, of low-protein intake on growth.^34,35^ In 2013, Hörnell et al.^34^ conducted a systematic review of studies performed on a broad range of age (0–18 years of life). In 2019, Pimpin et al.^35^ performed a meta-analysis on the effect of protein intake on growth outcomes, including weight and length gain, but pooled data at any time point from 0 to 77 months of age. In 2016, Patro-Golab et al.^36^ included in their systematic review interventions up to 3 years of life and provided separate meta-analyses on growth outcomes at 3 months, between 3 and 6 months of age, and between 6 and 12 months, with a range of included articles from 2 to 4 for each meta-analysis. The authors observed a lower mean length at 3 months of age among infants receiving low- vs high-protein formulas.^36^ However, the remaining analyses on several other outcomes, including weight gain, provided inconclusive results; in addition, the more recent literature included in our current analysis could not be considered by these authors.^36^ Finally, a recent publication by Stokes et al.^37^ observed similar results to those of Patro-Golab et al.^36^ This study included cohort studies only and the corresponding meta-analysis pooled data on outcomes collected during the first 2 years of life.

Differently from the previous systematic reviews and meta-analyses,^35–37^ a systematic review published in 2015^38^ achieved conclusions in line with ours; however, the heterogeneity of the considered studies was likely higher than in our analysis, because it included also studies on infants that were previously breastfed for >30 days or infants from obese mothers. Taken together, these analyses suggest that the effect of protein intake from formulas during the first year of life of full-term infants, if any, is likely limited, although the hypothesis of a stronger effect later in life cannot be discarded. This hypothesis is also supported by a recent very large epidemiological study, which suggested 2–6 years of life to be a possible critical age range to develop sustained obesity.^39^

The peculiar approach of our analysis relies on strict inclusion and exclusion criteria. We limited the meta-analysis to studies providing formula-based interventions with different protein content. Among them, we excluded those enrolling participants after 30 days of life. This choice was meant to reduce the possible confounding effect of previous prolonged breastfeeding. Exclusive breastfeeding has, indeed, an important impact on body growth. Breastfed infants present a higher body weight in the first 6–8 months of life, as compared to exclusively formula-fed ones,^40^ reaching values of ingested milk close to their plateaux as early as 1 month of age.^41^ Despite a further decrease in body weight through the first year, breastfed infants maintain a higher percentage of fat mass up to the third trimester of life.^42,43^

Following the previous argument, results of the three papers based on CHOP trial—still recruiting infants from 1 to 2 months of life (25% of infants were older than 30 days of life at enrollment)—were not considered in the current meta-analysis. In addition, as no information was available at 4 months, we could not materially include the papers by Koletzko et al.^20^ and Escribano et al.^21^ in the meta-analysis.

Most studies provided a modified casein/whey ratio in formulas. It is assumed that breastmilk is whey-predominant with an estimated mean ratio between whey and casein of 60:40. However, recent data obtained from individual measurements of casein-subunits’ concentrations suggest that this ratio changes throughout the lactation period.^44^

This study has strengths. No subject included in the meta-analysis was weaned, thus reducing the effect of possible additional dietary factors. Almost all studies were of good or fair quality and those included in the meta-analysis showed a low risk of bias. However, our analysis has limitations. The systematic review still included 12 articles only. The corresponding studies were mainly conducted in Europe and the United States; this could be due to our choice of restricting the systematic review to English papers, which has likely limited our ability to describe early nutrition somewhere else. In addition, the included papers had different objectives, with consequences in terms of eligible infants, sample size, type of protein-based intervention, and growth outcomes. The presence of many growth outcomes and the different timepoints for measurements have made the overall picture scattered and have prevented from drawing conclusions even on single growth outcomes, although the identified period of investigation was reasonably short. Among the 12 selected studies, we focused our meta-analyses on the effect at 120 days. As our meta-analysis included five studies only, derived evidence is limited. Evidence is also fraught with the moderate-to-high heterogeneity of included studies, partly due to a possible effect of previous breastfeeding among infants later enrolled in the formula arms. This heterogeneity, the small effects and related limited power found at the single study level, as well as the few studies included, are reflected in the low power of our meta-analyses to detect a difference between low- and high-protein content formulas. With few studies, it was also impossible to assess the presence of small study effects, including publication bias. However, based on a survey of meta-analyses published in the Cochrane Database of Systematic Reviews, tests for funnel plot asymmetry should be used in only a minority of meta-analyses.^33,45^ Finally, with such a few studies, we could not perform any subgroup analyses to assess the potential effect of relevant covariates, including study quality or sponsorship.

In conclusion, our systematic review does not allow us to conclude if different amounts of protein intake derived from either infant formulas or follow-on formulas during the first year of life provide an effect on growth outcomes in full terms infants; available evidence from our meta-analysis does not support the assumption that a differential protein content in formulas during exclusive milk-feeding leads to differences in growth outcomes at 4 months of life. Furthermore, properly powered studies including infants from birth and comparable outcomes measured at the same timepoints would allow us to definitively confirm the results of the present analysis. In the meanwhile, it is crucial to follow up formula-fed infants’ growth and provide evidence-based nutritional advice especially after weaning.

## Supplementary information

Supplementary materials and methods

## Data Availability

The datasets generated during and/or analyzed during the current study are available from the corresponding author on reasonable request.
