# Supplementary material for: Protein and growth during the first year of life: a systematic review and meta-analysis
Source: Pediatr Res. 2023 Mar 20;94(3):878–91. doi: 10.1038/s41390-023-02531-3 (PMC10444617; doi:10.1038/s41390-023-02531-3)

Manuscript: “Protein intake during the first year of life and anthropometric development in healthy term infants: a systematic literature review and meta-analysis”

Gregorio P. Milani and Valeria Edefonti
Online Supplementary Material

Supplemental materials and methods

Meta-analysis

Treatments

The paper by Oropeza-Ceja and coauthors[16] was based on a four-arm design, where the low-, medium-, and high-protein arms were considered, together with breastfeeding. In accordance with our definition, we included the original low (1.4 g/100 Kcal; 17 subjects) and medium (1.8 g/100 Kcal; 18 subjects) protein content groups into one low/medium-protein-content group of 35 participants.
The mean of the growth outcomes in the low/medium-protein-content group was calculated as the weighted mean of each outcome in the original low- (1.4 g/100 Kcal; 17 subjects) and medium- (1.8 g/100 Kcal; 18 subjects) protein content groups, with weights equal to the relative frequencies of subjects within each group (17/35 and 18/35, respectively).
The standard deviation of the growth outcomes in the low/medium-protein-content group was calculated using the decomposition of total variance in between- and within-groups variance. In detail, the total variance of each growth outcome was calculated as the sum of the within-groups and between-groups variances. Its standard deviation was obtained from the square root of the total variance. The between-groups variance was calculated as the weighted sum of the squared differences between each original mean (either low- or medium-protein content group) and the new low/medium-protein-content group mean of each growth outcome, where the weights were the two sample sizes. The within-groups variance was calculated having at the numerator the sum of the two original variances of growth outcomes in the low- and medium-protein content groups, weighted by each sample size, and at the denominator the total sample size minus the total number of groups (i.e., 35 - 2).

Statistical analysis

We used the mean difference (MD) in each growth outcome as the measure of treatment effect. We calculated the summary estimates of the weighted MD using both fixed- and random-effects models. In the former approach, we assume that every study is evaluating a common treatment effect and we considered the inverse variance method; in the latter approach, we assume that the effects observed in the studies are a random sample derived from a distribution of treatment effects, we were allowed to model both within- and between-study variation, and we used the DerSimonian and Laird estimation method[27,28]. We finally presented MDs from random-effects models because they provided a more conservative estimate[29]. In detail, we presented study-specific and combined estimates of the MD within a forest plot. Results from each study were

displayed in the following way: 1. a square represented the study-specific point estimate of the intervention effect, and: 2. a horizontal line represented the precision of the point estimate in the form of the confidence interval (CI). The area of the square reflected the contribution of each study to the meta-analysis in the form of a weight. The combined random-effects estimate and its CI were represented by a diamond[29].

Statistical heterogeneity among studies was assessed via chi-squared test on the Q statistic (results were defined as heterogeneous when the corresponding p-value is less than 0.10)[28]; the presence of potential inconsistencies was quantified through the I^2 statistic, which describes the percentage of total variation across studies that is due to heterogeneity rather than to chance[30]; values of the I^2 statistic <25% usually indicate low heterogeneity, those ranging between 25% and 75% moderate heterogeneity, and those >75% high heterogeneity.

For each trial, we plotted the treatment effect by its standard error within the funnel plot. Besides visual inspection, the presence of a symmetry in the funnel plot was assessed with statistical testing, including results from the Egger's and Begg's tests, to assess if the effect decreased with sample size increasing[31,32].

A cumulative meta-analysis was also performed to assess how the overall estimate changes as each study is added to the pool of the previously published studies.

Finally, we conducted an influence analysis by excluding each study at a time from the meta-analysis. The limited number of studies included in the meta-analysis did not allow to carry out the subgroup analyses by grade and type of sponsorship originally planned in the protocol. All the statistical analyses were performed using STATA software (version 13; StataCorp, College Station, TX, USA).

Supplemental References

16 Oropeza-Ceja LG, Rosado JL, Ronquillo D, et al. Lower Protein Intake Supports Normal Growth of Full-Term Infants Fed Formula: A Randomized Controlled Trial. *Nutrients* **10**, (2018).

27 DerSimonian, R. & Laird, N. Meta-Analysis in Clinical Trials. *Control Clin Trials* **7**, 177-188 (1986).

28 Greenland, S. Quantitative Methods in the Review of Epidemiologic Literature. *Epidemiol Rev* **9**, 1-30 (1987).

29 Harris, R. J., Bradburn M. J., Deeks J. J., Harbord R., Altman D. J., & Sterne J. A. C. Metan: Fixed and Random-Effects Meta-analysis. *The Stata Journal* **1**, 3-28 (2008)

30 Higgins, J. P., Thompson, S. G., Deeks, J. J. & Altman, D. G. Measuring Inconsistency in Meta-Analyses. *BMJ* **327**, 557-560 (2003).

31 Egger, M., Davey Smith, G., Schneider, M. & Minder, C. Bias in Meta-Analysis Detected by a Simple, Graphical Test. *BMJ* **315**, 629-634 (1997).

32 Thornton, A. & Lee, P. Publication Bias in Meta-Analysis: Its Causes and Consequences. *J Clin Epidemiol* **53**, 207-216 (2000).

Supplemental Figure 1: Funnel plot with pseudo 95% confidence limits for the random-effects meta-analysis comparing the effect on ***weight gain*** of high- vs. low-protein intakes in infant formulas. The treatment effect is plotted (x-axis) against its standard error (y-axis), here intended as the best available measure of precision. Each dot represents a study. A symmetric inverted funnel shape arises from a 'well-behaved' data set, in which publication and other forms of bias are unlikely.

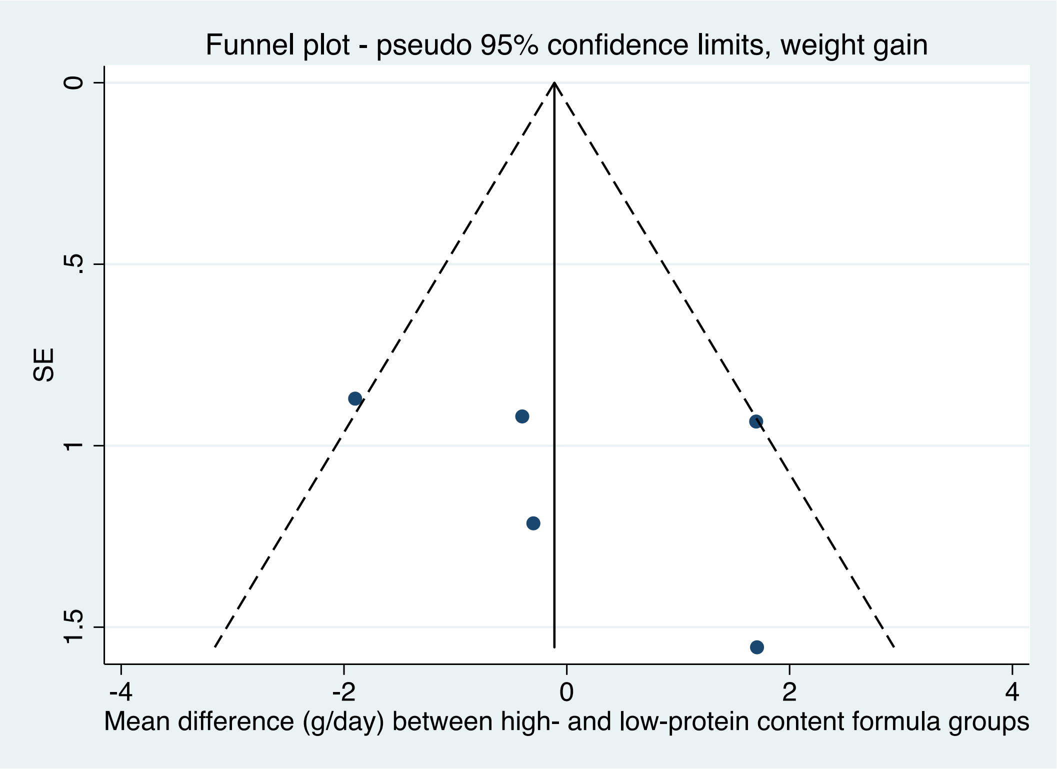

Supplemental Figure 2: Funnel plot with pseudo 95% confidence limits for the random-effects meta-analysis comparing the effect on ***length gain*** of high- vs. low-protein intakes in infant formulas.

The treatment effect is plotted (x-axis) against its standard error (y-axis), here intended as the best available measure of precision. Each dot represents a study. A symmetric inverted funnel shape arises from a 'well-behaved' data set, in which publication and other forms of bias are unlikely.

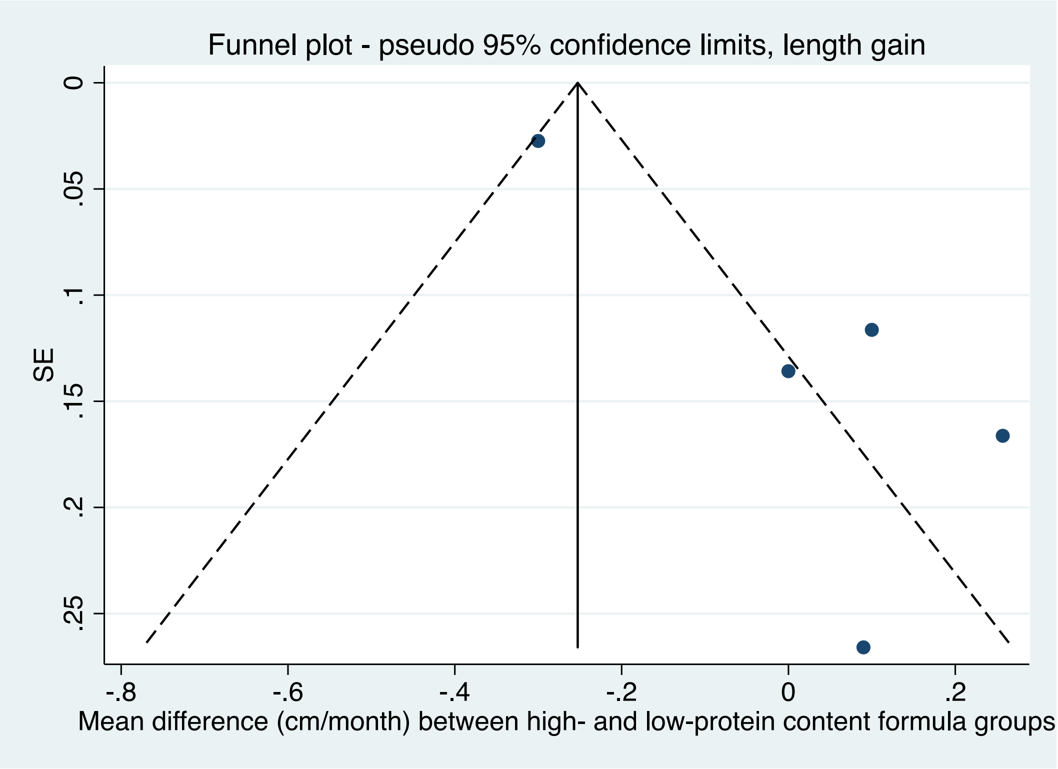

Supplement: Supplementary file 1 — Supplementary materials and methods [file 41390_2023_2531_MOESM1_ESM.pdf]
